# Biomechanical comparison of cemented versus cementless fixation in intraoperative femoral fractures during total hip arthroplasty

**DOI:** 10.1007/s00402-025-05987-6

**Published:** 2025-07-12

**Authors:** Ryunosuke Watanabe, Tomofumi Nishino, Tomohiro Yoshizawa, Fumi Hirose, Shota Yasunaga, Koshiro Shimasaki, Hajime Mishima

**Affiliations:** Department of Orthopaedic Surgery, Institute of Medicine, University of Tsukuba, Tsukuba, Japan

**Keywords:** Total hip arthroplasty, Intraoperative periprosthetic femoral fractures, Cerclage wiring, Biomechanical testing, Cementless stem, Cemented stem

## Abstract

**Introduction:**

Intraoperative periprosthetic femoral fractures (IPFF) are serious complications of total hip arthroplasty (THA) that occur more frequently with cementless stems than with cemented stems. Although cerclage wiring is commonly used to stabilize IPFFs, the biomechanical effects of subsequent stem fixation methods remain unclear. This study aimed to compare the biomechanical stability of cemented and cementless stems following cerclage wire fixation in an experimental IPFF model.

**Materials and methods:**

Six fourth-generation composite femurs with simulated Vancouver A2-type fractures were stabilized using a single 1.8 mm cerclage wire. Identical stem designs of polished cemented stems and fully hydroxyapatite-coated cementless stems were implanted in three specimens of each group. After applying a vertical axial load of 2000 N, an additional 40° of internal rotation was imposed. The primary outcome was the maximum torque at the time of fracture. Secondary outcomes included the maximum internal rotation angle, construct stiffness (calculated from force-displacement curves above 1500 N), and peak strain at three positions on the cerclage wire, measured using strain gauges.

**Results:**

The maximum torque was significantly higher in the cemented group compared to the cementless group (160.8 ± 24.9 vs. 89.1 ± 18.0 N·m, *p* = 0.016). Although the differences in stiffness and internal rotation angle were not statistically significant, both trended higher in the cemented group. The peak strain was consistently lower in the cemented group, with a significant difference at the medial side of the fracture line (*p* = 0.011, Cohen’s d = − 3.64).

**Conclusions:**

Cemented stems demonstrated superior biomechanical stability following cerclage wire fixation in the IPFF model, likely because of improved stress distribution via the cement mantle. Therefore, cemented fixation may be a favorable option for managing IPFF during THA.

## Introduction

Total hip arthroplasty (THA) is a widely performed procedure for managing advanced hip disorders, aiming to relieve pain and restore function. Both cemented and cementless femoral stems have demonstrated favorable long-term outcomes. However, complications such as infection, dislocation, and femoral fractures can adversely affect these results [1]. Intraoperative periprosthetic femoral fracture (IPFF) is one of the complications that occur during THA, with an incidence of 1.7–5.1%, and the risk of IPFF is higher with the use of cementless stems than with the use of cemented stems [2, 3]. The most common fracture site is the calcar, particularly during cementless stem insertion, where the proximal femur experiences the maximum stress [3–6]. If IPFF occurs, there is a higher risk of stem revision, postoperative complications, prolonged hospitalization, and increased medical costs [7, 8]. Therefore, the prevention and effective management of IPFF are critical for improving THA outcomes.

Among the fixation options, cerclage wire fixation is the most widely used method for commonly encountered calcar fractures [9–11]. Previous biomechanical studies have reported the effectiveness of cerclage wires [12–14]. Clinically, it is common to use an originally planned stem in combination with cerclage wiring when an IPFF occurs [11], making cementless stems a frequent choice even after IPFF. Although this approach may not always lead to complications, the initial rotational stability at the fracture site may be insufficient for cementless stems. In animal studies, Schutzer et al. found that bone ingrowth near the fracture site was inhibited despite cerclage wiring [15], potentially compromising the long-term mechanical stability. Cemented stems present challenges, such as the risk of impaired fracture healing because of cement leakage and reduced mechanical stability from suboptimal mantle formation. However, some reports suggest that cemented stems should not be avoided uniformly after IPFF [11], while others have shown no difference in stem survival rates between cemented and cementless stems [8]. Recent advances in implant design have allowed intraoperative conversion between cemented and cementless fixation using the same instrument, making this switch a more practical option. Therefore, in cases where IPFF occurs during cementless stem preparation, switching to a cemented stem after cerclage wire fixation may provide better initial fixation and stability than a cementless stem.

However, the biomechanical properties of cemented and cementless stems subjected to cerclage wire fixation have not been fully investigated. The choice of fixation after IPFF can influence complications such as stem subsidence and fracture progression, as well as patient satisfaction and functional outcomes [10, 16]. While previous studies have assessed cerclage wire techniques and biomechanical comparisons between different materials, direct comparisons between cemented and cementless fixations remain limited. Given the distinct load-transfer mechanisms, differences in biomechanical behavior under cerclage wire fixation are likely. Therefore, comparing the mechanical performance of cemented and cementless fixations using a clinically relevant cerclage wire fixation model may provide useful insights for implant selection when an IPFF occurs.

This study aimed to compare the biomechanical stability of cemented and cementless stems under cerclage wire fixation using an IPFF model. The load-to-failure strength and rotational stability of both fixation methods were measured to identify their differences. We hypothesized that cemented stems would demonstrate greater mechanical strength than cementless stems, even after wire fixation. Our findings aim to provide objective data to inform clinical decision-making in cases of IPFF.

## Materials and methods

### Materials and femoral preparation

This study used fourth generation composite femurs (Model 3403; SawBones, Pacific Research Laboratories, USA) were used in this study. These composite femurs are made of glass-fiber-reinforced epoxy and are designed to replicate the mechanical properties and anatomy of an adult male femur (183 cm, 890 N [90.3 kgf]). The structure consists of a cortical bone shell and a cancellous bone core [17, 18].

The implants used were Universia stems (TEIJIN NAKASHIMA MEDICAL, Japan), comprising a full hydroxyapatite (HA)-coated cementless stem and a polished cemented stem, both with identical geometry. Only the collared type was available for the cementless stem; therefore, a collared cementless stem was used. In contrast, the cemented stem lacked a collar. The cemented version follows the line-to-line concept, with fixation achieved by close cortical contact with minimal cement mantle [19]. Stem sizing was determined using 3D templating software (ZedHip, version 17.0.0, Lexi Co., Ltd, Japan). A size 11 high offset cementless stem was selected to preserve a thin cancellous bone layer between the stem and the cortical bone. A size 10 high offset cemented stem, which was one size smaller, was chosen because the cancellous bone of the composite femur was dense and did not permit cement penetration, requiring maintenance of a thinner cement mantle around the stem. This size difference provided an approximate reduction of 0.5 mm in the anteroposterior dimension and 1.0 mm in the mediolateral dimension, compared with the reductions achieved with the size 11 cementless stem. PALACOS R (Heraeus Medical, Germany) was used as the bone cement. The cerclage wire was a Ti-6Al-4 V alloy wire with a 1.8-mm diameter (Wiring System, TEIJIN NAKASHIMA MEDICAL, Japan).

**Fig. 1:**
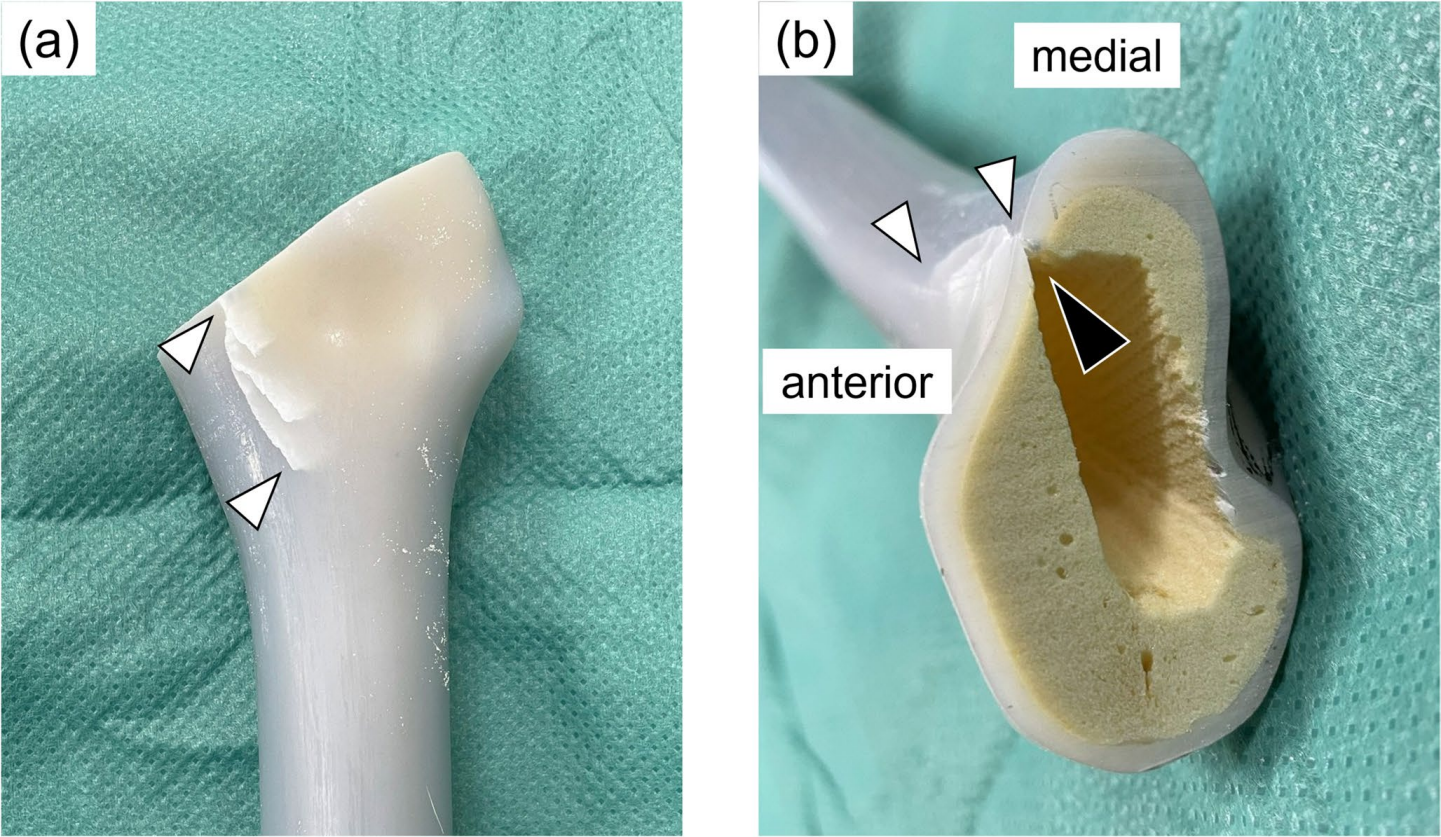
Creation of the intraoperative femoral fracture model. (a) An oblique crack was created at the calcar region using a chisel, simulating a Vancouver type A2 fracture (white arrowheads). (b) Cross-sectional image of the proximal femur showing the fracture line (white arrowheads). The fracture was initiated by inserting a chisel at the anteromedial corner of the femoral canal (black arrowhead)

**Fig. 2:**
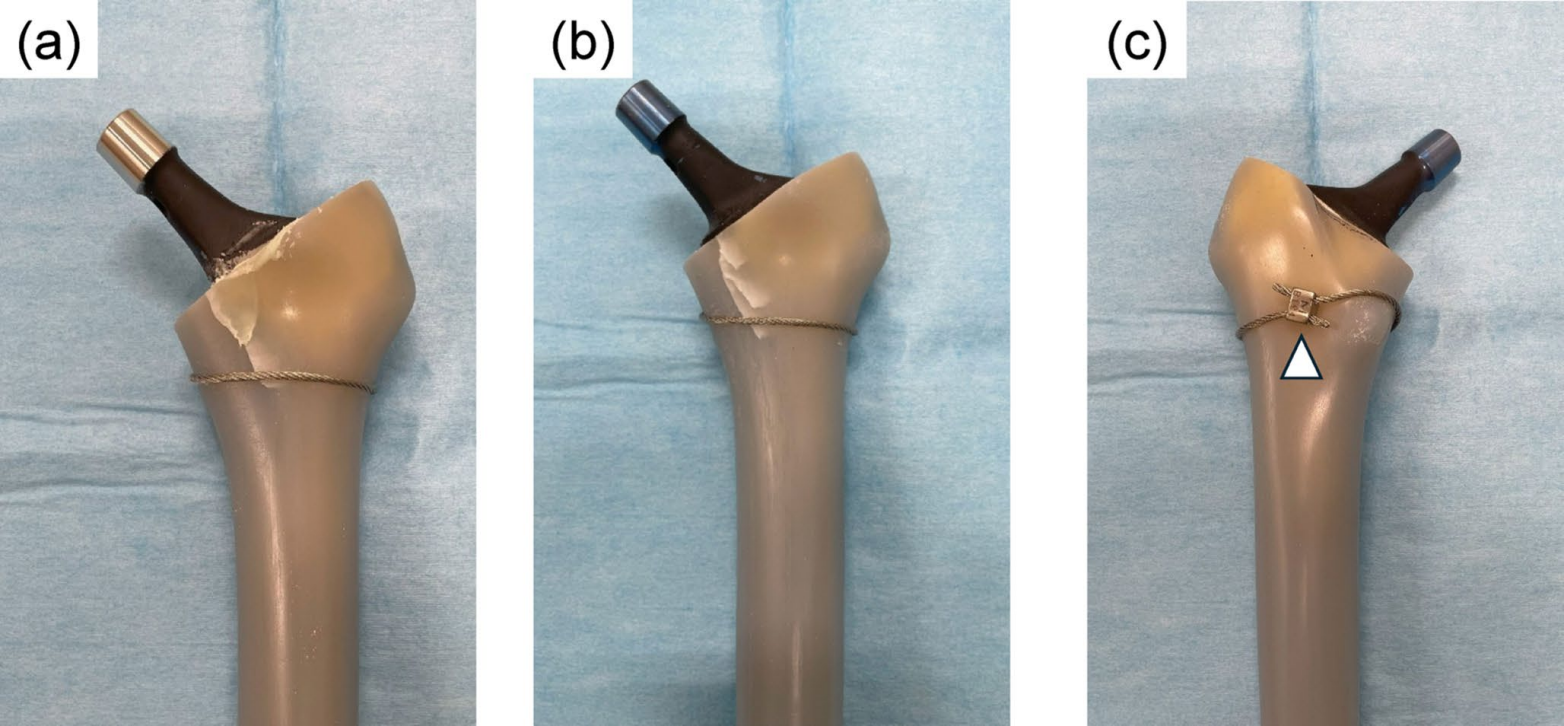
Stem implantation and cerclage wire fixation in the fracture model. (a) A cemented stem was inserted after fracture creation and wire fixation. (b) A collared cementless stem was inserted, with the collar firmly contacting the calcar region

Six composite femurs were used in this study. Following standard THA procedures, the femur was cut 10 mm proximal to the lesser trochanter using an oscillating saw, and the medullary canal was broached to a size 11. A calcar reamer was not used because the osteotomy was performed at an angle matching the top surface of the broach. To create a calcar fracture, a thin chisel was inserted into the anteromedial corner of the canal [14]. This method provided a reproducible and stable fracture model (Fig. 1). A fracture line was created 25–35 mm distal to the osteotomy line to simulate a Vancouver A2 type fracture [20]. A single 1.8-mm wire was wrapped around the femur at the level of the lesser trochanter and tightened to 30 N. A wire clamp was positioned 20 mm posterior to the lesser trochanter apex. Subsequently, a femoral stem was inserted into the canal. For the cemented stem, a distal plug was inserted, and bone cement was applied using a third-generation technique involving retrograde insertion via a cement gun and pressurization. A size 10 stem was then implanted at the same depth as that of the broach. For the cementless stem, size 11 was impacted until the collar contacted the calcar. (Fig. 2). Three femurs were assigned to each group (cemented and cementless). To ensure consistency, all procedures were performed by a single orthopedic surgeon with 10 years of clinical experience.

### Mechanical testing

**Fig. 3:**
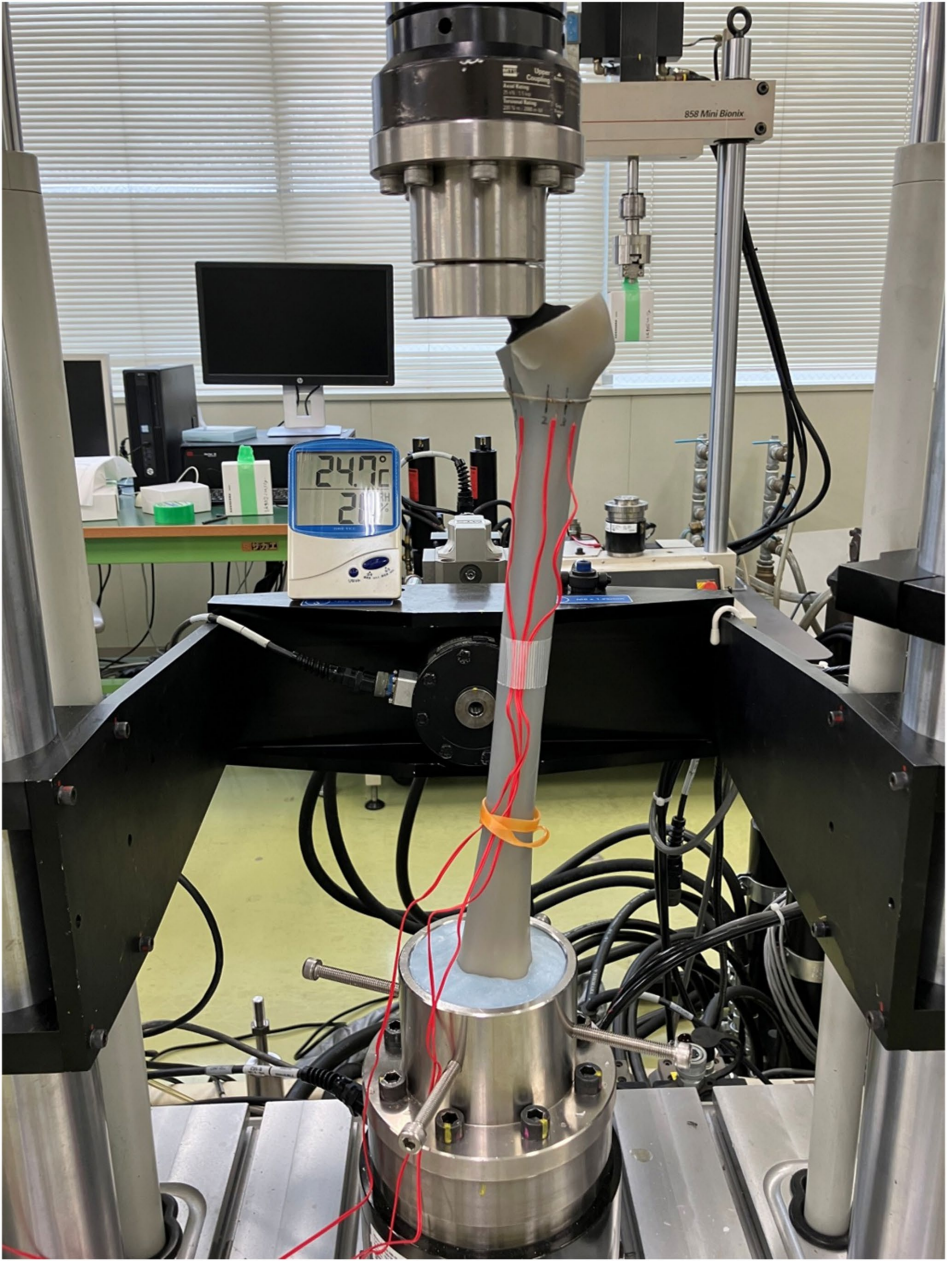
Experimental setup for mechanical testing. Photograph showing the femur model mounted on the mechanical testing system. Strain gauges were attached directly over the cerclage wire at three locations to measure the surface strain during loading

**Fig. 4:**
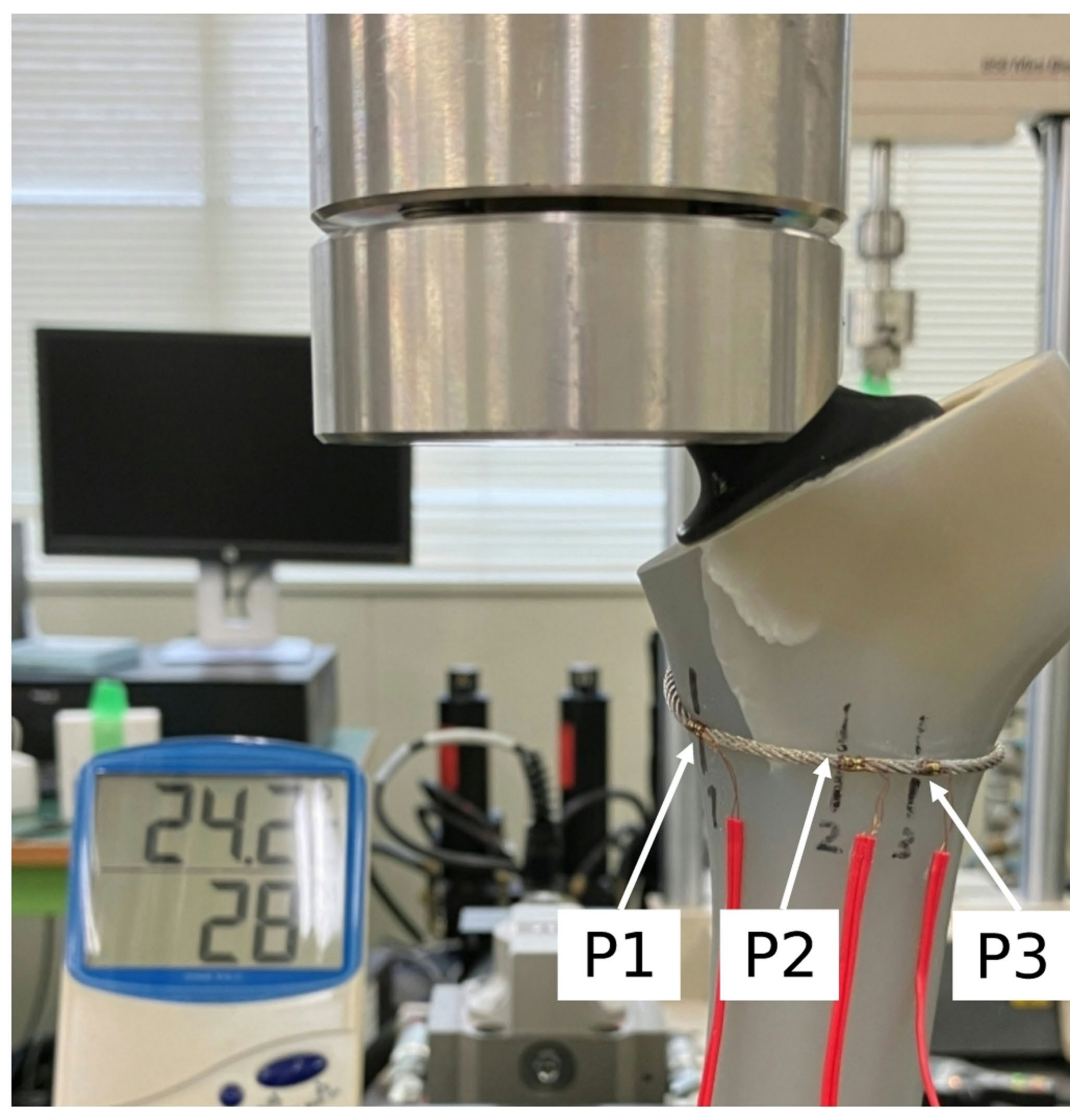
Locations of strain gauges on the cerclage wire. Strain gauges were attached directly to the cerclage wire at three positions: P1 (1 cm medial to the fracture line), P2 (1 cm lateral), and P3 (2 cm lateral)

Each femur was mounted on a testing machine (Bionix^®^, MTS, USA) with the center of the femoral head and the condylar centers aligned to the vertical axis in the coronal plane. In the sagittal plane, the tabletop surface passing through the posterior condyles and posterior edge of the greater trochanter was set perpendicular to the floor. The distal femur was fixed in a pot using bone cement (Fig. 3). Three uniaxial strain gauges (uniaxial, gauge length 0.2 mm; KFG-02-120-C1-11L3M2R, Kyowa, Japan) were attached to the cerclage wire at the following locations: 1 cm medial to the fracture line (P1), 1 cm lateral to the fracture line (P2), and 2 cm lateral to the fracture line (P3) (Fig. 4). The strain was measured using a PCD300A system (Kyowa, Japan).

The loading conditions were similar to those in previous studies. A vertical load of 2000 N was applied for 10 s and then maintained. While under load, the femur was internally rotated by 40° within 1 s to induce a fracture [21, 22].

### Outcomes and statistical analysis

The primary outcome was maximum torque at the point of fracture. The secondary outcomes included the maximum internal rotation angle at the fracture, axial stiffness (N/mm) during vertical loading, and peak strain just prior to fracture, as measured by the strain gauges. The stiffness was calculated using regression analysis as the slope of the displacement curve between 1500 and 2000 N of the applied load.

Levene’s test and Student’s t-test were used to compare the cemented and cementless groups. A significance level of 0.05 was adopted, and the effect size was calculated using Cohen’s d, with d ≥ 0.8 considered a large effect. Statistical analyses were performed using SPSS version 29.0 (IBM Corp., Armonk, NY, USA).

## Results

**Fig. 5:**
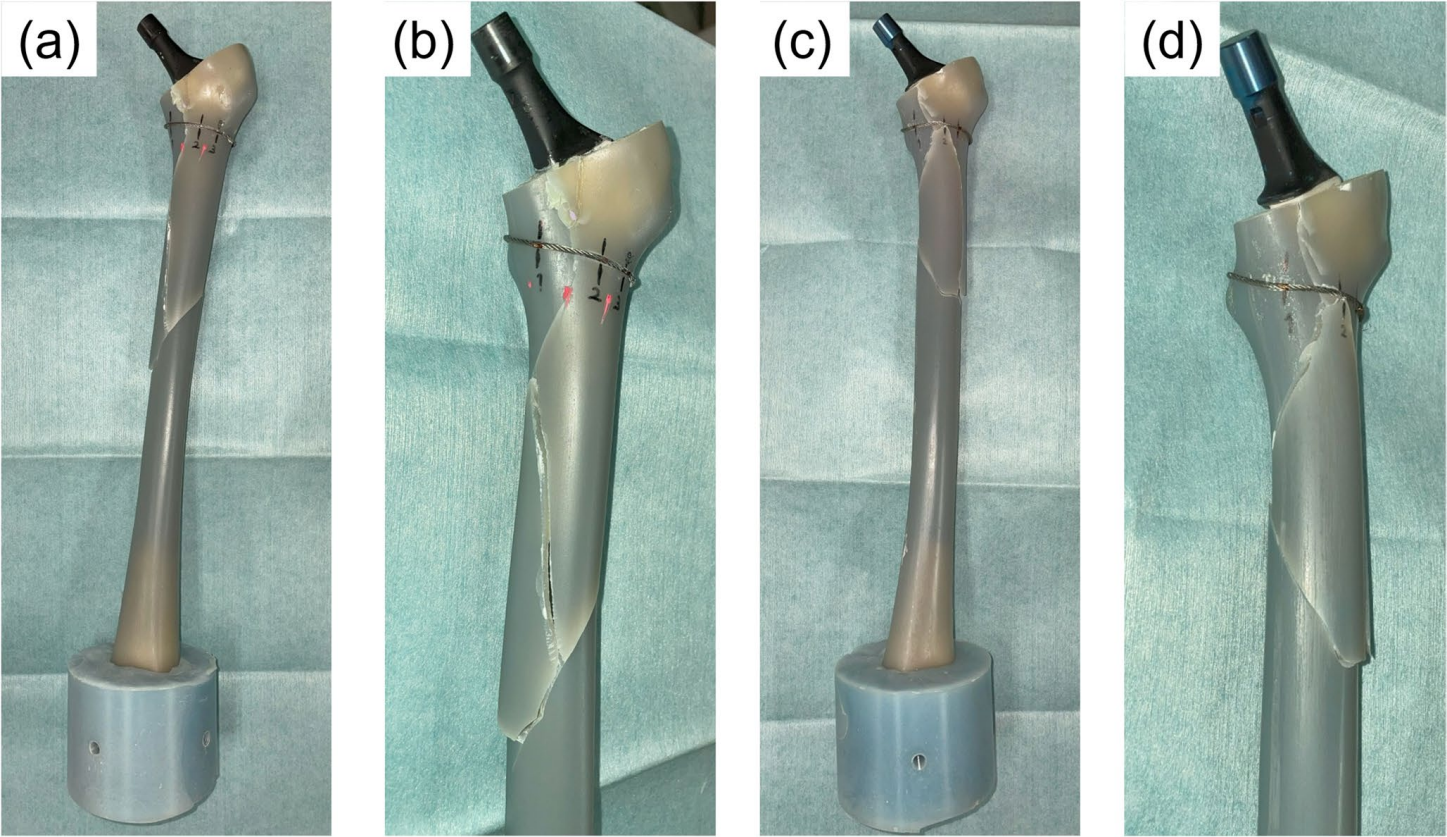
Representative fracture patterns observed after torsional load-to-failure testing. (a, c) Overview images. (b, d) Anterior–medial views. (a, b) Cemented stem group: a spiral fracture can be seen originating at the calcar and extending distally along the medial cortex. (c, d) Cementless stem group: a spiral fracture can be seen originating at the calcar and propagating distally along both the medial and lateral cortices. The collar is displaced from the calcar following fracture

Fractures occurred in all six composite femurs. In the cemented group, one specimen sustained a fracture just above the distal fixation pot at an internal rotation of 39.7°. The other two specimens showed a distal extension of the calcar fracture, followed by a spiral fracture pattern. In the cementless group, all three specimens were fractured in the proximal region. Two of these displayed distal propagation of the calcar fracture, resulting in spiral fractures similar to those observed in the cemented group (Fig. 5). In the remaining cementless specimen, the fracture originated at the anterior portion of the trochanteric osteotomy line, located anterior to the calcar fracture, and progressed distally in a spiral pattern.

The primary outcome, maximum torque at the point of fracture, was significantly higher in the cemented group (160.8 ± 24.9 N·m) compared to the cementless group (89.1 ± 18.0 N·m; *p* = 0.016, Cohen’s d = 3.30) (Table 1). Although not statistically significant, the cemented group also tended to have higher maximum internal rotation angle stiffness values above 1500 N.

**Table 1:** Summary of mechanical test results

	Cemented stem	Cementless stem	*p*-value	Cohen’s d
Maximum torque (N・m)	160.8 ± 24.9	89.1 ± 18.0	**0.016**	3.3
Maximum internal rotation angle (°)	31.3 ± 7.8	20.8 ± 4.6	0.115	1.6
Stiffness (N/mm)	3221.7 ± 472.2	2224.4 ± 1303.9	0.281	1.0
P1 strain	807.8 ± 630.7	3759.8 ± 957.4	**0.011**	3.6
P2 strain	1858.9 ± 1663.1	2033.0 ± 2077.0	0.915	0.1
P3 strain	2480.5 ± 591.2	2651.7 ± 935.5	0.802	0.2

Mean ± standard deviations are shown for each outcome measure. P-values and Cohen’s d effect sizes are provided for comparisons between the cemented and cementless stem groups. Statistically significant differences (*p* < 0.05) are highlighted in bold

The cemented stem group demonstrated significantly higher maximum torque and significantly lower strain at P1 compared to the cementless group. Other measures showed no statistically significant differences

Strain values measured at the three positions on the cerclage wire (P1, P2, and P3) were consistently lower in the cemented group than in the cementless group. The largest strain difference between groups was observed at P1 (1 cm medial to the fracture line), with a statistically significant reduction in the cemented group (807.8 ± 630.7 vs. 3759.8 ± 957.4, *p* = 0.011). In contrast, strain differences at P2 and P3 were smaller and not statistically significant (P2: *p* = 0.915; P3: *p* = 0.802) (Table 1).

## Discussion

This study compared the mechanical properties of cemented and cementless stems implanted into femoral models following cerclage wire fixation for IPFF. The results showed that the maximum torque at the time of fracture was significantly higher in the cemented group. The maximum strain measured on the wire was lower in the cemented group at all measurement points, with a significant difference observed at the site medial to the fracture site. Additionally, the cemented group showed a trend toward a greater internal rotation angle and stiffness, although these differences were not significant. These findings suggest that cemented stems may provide enhanced rotational resistance and structural stability, even after fracture fixation.

These results are consistent with those of previous studies showing that cemented stems with geometries identical to those of cementless stems yielded higher failure loads [23]. In this study, polished cemented stems and fully HA-coated cementless stems with identical geometries were tested in cadaveric femurs without fractures. The cemented stem achieved an average failure load of 2.8 kN, whereas the cementless stem reached 2.2 kN, indicating an approximately 25% higher failure load with cement fixation than cementless stem. Although the present study used composite femurs with stimulated periprosthetic fractures stabilized with cerclage wire, a similar difference exceeding 25% in maximum torque was observed, and the stiffness values were likewise higher in the cemented stems. These findings support the notion that cement fixation may contribute to increased structural stability of the femur.

Strain gauge measurements further indicated that the cemented stems consistently exhibited lower strain on the cerclage wire, particularly at the medial aspect of the fracture (P1), where the difference reached statistical significance. This may be explained by the load-dispersing effect of the cement mantle, which could reduce localized stress and mitigate fracture propagation. Previous studies have also reported that cemented stems significantly reduce bone surface strain in the proximal medial femur compared to cementless stems [24], reinforcing the biochemical rationale for cemented fixation in the context of IPFF.

Although this study did not include a control group of unfractured composite femurs, reference to previous biomechanical studies conducted under similar loading conditions provides a basis for interpreting our results.

Morishima et al. reported that, in unfractured composite femurs implanted with Exeter stems, the median fracture torque ranged from 114.3 to 180.2 Nm depending on the stem length and offset [21]. Similarly, Windell et al. compared three types of cemented stems (Exeter, CPT, and C-stem) in unfractured composite femurs and reported mean fracture torque values ranging from 124 to 174 Nm [22]. In our IPFF model with cerclage fixation, the cemented stem group demonstrated a mean torque of 160.8 ± 24.9 Nm, which was within the range reported in these previous intact models. These findings suggest that cerclage fixation may partially restore rotational stability following fracture, and despite the absence of a control group including healthy bones, they somewhat support the mechanical relevance of the present fracture model.

The collar of a cementless stem contributes to rotational stability and reduces the risk of periprosthetic fracture [25–28]. The Universia cementless stem used in this study features a collared design, which may have contributed to the mechanical stability observed in the cementless group. The observed differences in maximum torque and strain between the cemented and cementless groups may have been greater if a collarless cementless stem had been used. The collar likely enhanced the stability of the cementless stem, partially reducing the mechanical differences compared to the cemented stem.

The results of this study indicate that cemented stems provide sufficient mechanical stability following IPFF, which is clinically significant. This is particularly important for older patients or individuals with osteoporosis, where immediate stability is essential to facilitate early mobilization. These results support the need to reevaluate the indications for cemented fixation in these groups. Previous reports have shown no significant differences in long-term stem survival rates between cemented and cementless stems after IPFF, suggesting that both fixation methods can achieve good outcomes under appropriate conditions [8]. However, when selecting cemented fixation, care must be taken to avoid cement leakage through the fracture line, which can interfere with bone healing, and to ensure adequate mantle formation to maintain the fixation strength. Proper sealing of the fracture line and precise timing of cement application are necessary [11].

This study had some limitations. First, the small sample size limited the statistical power of the analysis. Second, the composite femurs used did not fully replicate the structural and material properties of human bones, particularly with respect to trabecular architecture and biological variability. Third, a control group of unfractured femurs was not included, and this limited the ability to directly quantify the mechanical impact of the fracture itself. In addition, only one type of cementless stem and one type of cemented stem design were evaluated, both of which had the same geometry. Furthermore, this study used only a single static loading test without assessing repeated loading or long-term stability. Future studies should include a larger number of specimens, different bone quality models, implant designs, and evaluations under multiple loading conditions to confirm the reproducibility. Additionally, inclusion of a control group of unfractured femurs would be important to directly assess the mechanical impact of intraoperative fractures and to validate fracture models more comprehensively.

## Conclusion

This study evaluated the biomechanical performance of cemented versus cementless femoral stems inserted after cerclage wire fixation in an IPFF model. The results showed that the cemented stems demonstrated a significantly higher maximum torque at fracture and tended to produce lower strain on the wire compared to cementless stems. These findings indicate that cemented stems may be an effective option for implant selection after intraoperative fracture. Further studies incorporating different bone qualities, implant designs, and loading conditions are warranted to validate and extend these results in more clinically relevant contexts.

## Data Availability

No datasets were generated or analysed during the current study.
